# Assessing the efficacy of aspiration and ethanol injection in recurrent endometrioma before IVF cycle: A randomized clinical trial

**Published:** 2013-03

**Authors:** Abass Aflatoonian, Elham Rahmani, Mozhgan Rahsepar

**Affiliations:** 1 *Research and Clinical Center for Infertility, Shahid Sadoughi University of Medical Sciences, Yazd, Iran.*; 2 *Department of Obstetrics and Gynecology, Bushehr University of Medical Sciences, Bushehr, Iran.*

**Keywords:** *Endometrioma*, *Ethanol sclerotherapy*, *Fertilization In-vitro*

## Abstract

**Background: **Endometriosis is a common hormone-dependent gynecologic disease with a high recurrence. Laparotomy or laparoscopy is the standard surgery for the large endometrioma. Also, sclerotherapy is basically used to treat different diseases one of which is endometrioma.

**Objective:** The study was designed to assess the value of transvaginal ultrasound-guided ethanol sclerotherapy in patients with a recurrent endometrioma.

**Materials and Methods:**In a randomized clinical trial, an interventional group of 20 patients underwent transvaginal ethanol sclerotherapy for recurrent ovarian endometrioma. The patients were followed up first after one and two weeks and then after one, two, and three months. If the patients had no endometrioma, they were treated with in vitro fertilization (IVF) (standard long protocol). A control group of 20 patients with endometrioma were enrolled for an IVF protocol. They had no treatment by ethanol sclerotherapy. IVF parameters, pregnancy rates, and implantation rates were compared in both groups.

**Results:** The demographic data showed no difference between the two groups. The initial mean endometria size was 41.45±15.9 cm, the recurrence rate after 6 months was 4 (20%), FSH before and after sclerotherapy was 6.97±2.25 IU/L and 6.78±1.88 IU/L (p=0.343). The clinical pregnancy rate was 6 (33.3%) vs. 3 (15%), (p=0.616). The fertilization rate emerged 63.06% in study group vs. 60.38%, (p=0.57). The implantation rate turned out 12.9% in study group vs. 7.5%, (p=0.52). None of these results were significant. However, the data pointed to a better trend toward the ethanol sclerotherapy group.

**Conclusion:**Ethanol sclerotherapy could be an effective strategy for the treatment of recurrent endometrioma especially before IVF.

## Introduction

Endometriosis is a general estrogen-needy disorder with a significant recurrence rate (1). Several medical methods are available to treat endometriosis. The current evidence is insufficient to support the superiority of one treatment over another (2). However, medicinal treatment has proved to be of less benefit than surgical treatment for infertile women. On the other hand, ovarian surgery may reduce ovarian reserves in women with an advanced disease (3).

The surgical removal of endometrioma may cause not only the removal of healthy ovarian tissue but also surgery-related local inflammation or vascular compromise, which may have a harmful effect on the residual primordial follicle pool. Actually, the bilateral removal of endometrioma by laparoscopy caused a premature ovarian failure at a 2.4% risk (Busacca *et al* 2006). Reportedly, this is one of the reasons why sclerotherapy is another option beside laparotomy or laparoscopy. Researchers have shown that the surgical treatment of endometrioma has no considerable impact on in vitro fertilization (IVF) pregnancy rates and ovarian response after stimulation (4). Juan *et al* recommend keeping on straight to IVF to decrease time of pregnancy, to diminish surgical complications and to lower the costs. Surgery should be considered only for large cysts or painful disorders which are intractable to medical handlings, or once malignancy cannot consistently be excluded (5). One of the other treatments for endometrioma is ultrasound-guided aspiration. After a simple aspiration, the recurrence rate has been up to 100%; however, repeated aspiration of endometrioma is another suggestion (6). 

Studies have reported that a combination of aspiration and sclerosing agent could be effective at reducing the recurrence rate (7-10) on the other hand, Kafali *et al* studied high recurrence rate in cyst with a bloody aspirate and high CA125 levels in cyst fluid (11). It seems that the precision of transvaginal ultra sonography is acceptable for discovering the type of ovarian cysts without any fright from malignancy or complication (12). 

Another application of sclerotherapy is for simple cysts that are painful or liable to torsion. Such patients, who are at a low risk of malignancy, are relieved with sclerotherapy while avoiding surgery (13). Moreover, the aspiration of basal cysts does not improve ART outcome when E_2_ levels are suppressed (14). Ethanol retention in chocolate cysts guided by ultrasound is a therapeutic approach with a high cure rate. Some researchers have pointed out that ethanol is left for five to ten minutes. Also, some others have reported that effective sclerotherapy occurs, when ethanol is retained in the endometrioma (8, 9, 15-19). 

In this study, we comparatively investigated the IVF outcome in the recurrent endometrioma with and without ethanol sclerotherapy and the recurrence rate after alcohol treatment.

## Materials and methods

**Figure 1:**
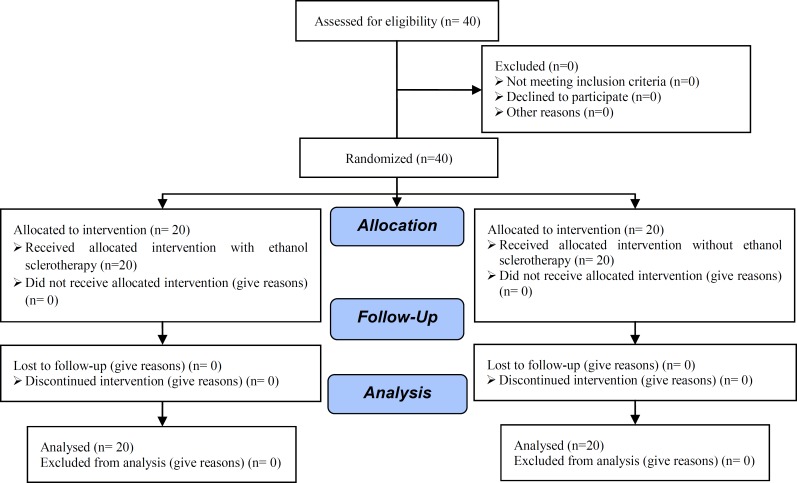
Consort flow diagram.

In a prospective randomized clinical trial (figure 1), 40 patients participated between March 2011 and February 2012 after they provided a written informed consent. This study was approved by the Committee of Ethics of Yazd Research and Clinical Center for infertility affiliated to Shahid Sadoughi University of Medical Sciences. Randomization was done, besides using computerized randomization, by opening sealed envelopes. 

The inclusion criteria were FSH<10, 20<age<39 years, past history of operation on endometrioma which recurrent unilateral endometrioma was occurred again and recurrent endometrioma more than 30 mm. The exclusion criteria were liver or kidney or heart diseases and sever male factor infertility. The diagnosis of endometrioma was based on sonography and the result of previous laparotomy/laparoscopy.

Twenty patients, assigned as the interventional group, received transvaginal sclerotherapy for ovarian endometrioma. In the operation room, the patients received pentazocin (Ampulla, 30 mg/ml, Tolidaru, Iran) and pethedine (Ampulla, 50 mg/ml, Kaspian, Iran) as analgesic agents. Cefixim 400 mg/day was started as a prophylactic antibiotic before and after sclerotherapy for one week. A needle (three-way needle, Wallace, UK) was introduced into the endometrioma and aspiration was done under sonography guidance (Honda 4000, Japan).

The endometrioma was irrigated with a sterile normal saline till the fluid was clear in color. The endometrioma was filled with 98% ethanol for 10 minute and at the end ethanol was aspirated. The volume of ethanol was calculated 80% of the aspirated endometrioma fluid .Then fluid was sent for a cytological test for all the patients. Then the patients were followed up after one week, one, two, and three months. 

After three months, if the patients had no complication or endometrioma, along IVF protocol was started. The IVF protocol was started for the control group without sclerotherapy. All the patients underwent pituitary desensitization (FSH ≤5 IU/ml, LH ≤5 IU/ml, Estradiol ≤50 pg/ml, and progestrone≤1ng/ml) with suprefact (Buserelin acetate, Aventis Pharma Deutschland, Germany) at a dose of 0.5µg/day initiated in the mid luteal phase. It continued after menstruation at a dose of 0.25µg/day until the day of U-HCG administration. 

Gonal-F (Gonal-F, Serono, Italy) was initiated from the second day of menses according to the patient's age and antral follicular count. Then, the dose of Gonal-F was adjusted for follicular monitoring by sonography and estradiol. First, sonography was done on the seventh day of menstruation and repeated as needed .When the follicular size reached 18-20 mm, HCG (pregnyl, Daropakhsh, Iran)10000 IU was administered. After 34-36 hours, a transvaginal ultrasound-guided ovum pick-up was performed. 

Two fresh embryo transfers were performed on the third day after ICSI with a Labotect catheter (Labotect, Gotting, Germany). The luteal phase was supported by Cyclogest (Actavis, Barnstaple, EX32 8NS, UK) vaginally 400 mg/ two times per day. It was started on the day after the oocyte retrieval. Serum βhCG was checked 14 days after the embryo transfer. If the patient was pregnant; Cyclogest was continued till the tenth week of gestation. Clinical pregnancy was confirmed by transvaginal sonography with at least one gestational sac in the uterine cavity. Recurrence would be anticipated during 6 months if there was any reappearance of endometrioma according to vaginal ultrasonography in non-pregnant patients. Finaly, IVF parameters, fertilization rates and implantation rates were compared in the two groups.

## Statistical analysis

Statistical analysis was performed with a SPSS software (version 16).The significance of differences was statistically checked by t-test, paired t-test, Mann-Witney and χ^2^-test. P<0.05 was considered statistically significant.

## Results

Forty patients who met the inclusion criteria of the study were randomly divided into two groups. The size of the endometrioma was 41.45±15.9 (cm). Malignant cells were not seen in the pathology report of aspirated endometrial fluid. No complication occurred in any patient. The endometrioma recurred in 4 (20%) patients after 6-months post sclerotherapy. 

No difference was seen between the two groups in terms of mean age, duration of infertility, FSH, LH, TSH and antral follicular count (AFC) (Table I). FSH was checked on the second day before (6.97±2.25 IU/ml) and 3 month after (6.78±1.88 IU/ml) sclerotherapy (p=0.343). The IVF outcome was compared in both groups (Table II). 

There were no significant differences with regard to the total dosage of gonadotropin (p=0.866), estradiol on the day of HCG (p=0.874), duration of gonadotropin consumption (p=0.553), total number of follicles (p=0.331), oocytes and embryos, number of transferred embryos, and Metaphase II oocytes (p=0.476). Good-quality embryos were achieved in 94.4% and 50% of the study group and the control group respectively (p=0.003). Chemical pregnancy, clinical pregnancy fertilization and implantation rate were described in the study and control groups as 6 (33.31%) vs. 4 (20%) p=0.468, 5 (27.8%) vs. 3 (15%) p=0.616, 63.06% vs. 60.38% p=0.57, and 12.91% vs. 7.5% p=0.52 respectively.

**Table I:** Baseline patient characteristics

	**Study group** **Mean ± SD**	**Control group** **Mean ± SD**	**p-value**
Age (years)	29.4 ± 5.76	31 ± 3.89	0.231
Duration of infertility (years)	5.05 ± 2.85	6.5 ± 2.81	0.072
FSH (IU/ml)	6.97 ± 2.25	6.64 ± 2.44	0.565
LH (IU/ml)	5.83 ± 2.18	6.03 ± 2.07	0.862
TSH (mIU/L)	2.53 ± 0.8	2.14 ± 0.64	0.149
Antral follicular count(number)	8.8 ± 1.6	8.25 ± 2.1	0.369

Mann-Witney and t-test, p-value <0.05 significant

**Table II:** Comparison of IVF outcomes in both groups

	**Mean ± SD** **Study group**	**Mean ± SD** **Control group**	**p-value**
Total dose of gonadotropin (IU)	2491.6 ± 662.6	2523.7 ± 499.4	0.866
Estradiol (On the Day of HCG)	1466.6 ± 1367.6	1280 ± 892.8	0.874
Duration of gonadotropin	8.94 ± 1.21	9.65 ± 2.25	0.553
NO. of Follicle	10.5 ± 3.88	9.4 ± 3.39	0.331
NO. of total oocyte	7.83 ± 2.22	7.55 ± 4.77	0.393
NO. of total embryo	4.72 ± 2.76	3.8 ± 2.56	0.319
NO. of embryo transfer	2.17 ± 0.7	2.35 ± 0.67	0.331
NO. of Metaphase II oocyte	6.11 ± 2.34	5.45 ± 3.18	0.476

NO= Number, Mann-Witney and t-test, p-value <0.05 significant, HCG=Human Chorionic Gonadotropin

**Table III:** Comparison of pregnancy results in both groups

	**Study group** **No (%)**	**Control group** **No (%)**	**p-value**
Chemical pregnancy	6 (33.3%)	4 (20%)	0.468
Clinical pregnancy	5 (27.8%)	3 (15%)	0.616
Fertilization rate	63.06%	60.38%	0.57
BMI (kg/m^2^)	25.00±3.2	24.30±3.6	0.557

NO=Number, Mann-Witney and t-test, p-value <0.05 significant

## Discussion

We found that ethanol sclerotherapy is a safe and alternative modality in the treatment of recurrent endometrioma. Ikuta *et al* used ethanol for 5 minutes and aspirated it all. The recurrence rate was 11.1% (16). Yazbeck *et al* left ethanol for 10 minutes, and the recurrence rate was 12.9% at a mean time of 10 months after sclerotherapy. In addition they demonstrated that the number of mature oocytes was higher in the ethanol group (9). Similarly, Salem *et al* reported ethanol sclerotherapy may improve IVF outcome (19). Hsieh *et al* mentioned the retention of ethanol in the endometrioma was more effective than the removal of alcohol. Hsieh reported the recurrence rate was 13.3% in retention of ethanol versus 32.1% when ethanol was removed during one year (8).

Zhu *et al* reported that the monthly repetition of aspiration could decrease the recurrence rate from 91.5% to 27.9% after two years (6). In our study, ethanol was left for ten minutes and the recurrence rate was 20% in 6 months, as in some other studies. Although there was no significant difference between the IVF outcomes of the two groups, some good results were achieved in favor of the ethanol group. It appears that the presence of bilateral endometrioma during IVF treatment is not associated with reduced embryo quality (20). Koike *et al* observed that the number of good-quality embryos was higher in the ethanol group(17). 

Also, we had significantly good-quality embryos in our study group. But Okagaki *et al* reported unusual adhesion in four patients who had experienced ethanol sclerotherapy which might have been reasoned by the leakage of endometrioma or ethanol (21). The epithelial cells lining the cyst walls play a critical function. When ethanol contacted the cyst walls, the coagulation cascade was activated and inflammatory mediators and fibrosis were produced. It was critical for the adhesion of the cyst walls and for the avoidance of reaccumulation of the endometrioma (13, 22). It has also been reported that ethanol treatment induces dehydration of the surrounding tissues, leading to a loss of function in these tissues (17). 

Chang and Hsieh reported a raise in the amount of antral follicles after ethanol sclerotherapy. When endometrioma was in the vicinity of follicles, it could make the oocyte retrieval more complicated and contaminate the follicular fluid with the content of the endometrioma. After sclerotherapy, the negative mechanical pressure of endometrioma was eliminated from the folliculogenic property of the ovary, and a free space was created for the dominant follicles. 

Moreover, the ovarian blood supply got better (8, 23, 24). In the endometrium, aromatase P450 is also stated under pathological forms, and local estrogen biosynthesis is considered to be essential to the pathophysiology of a diversity of uterine disorders, including fibroids, endometriosis, and adenomyosis (25). The decreased expression of HOXA10 may designate impaired endometrial receptivity in patients with endometriosis (26, 27). 

A probable limit of this study is associated to the period for the follow-up evaluation. It can be quarreled that a 3-6 month period is too short for recurrence. In contrast, endometriosis may progress over time. Therefore a longer follow-up could postpone ovarian stimulation. An attempt for conception as soon as possible after surgery seems advisable since waiting is tantamount to a lower pregnancy rate and a higher rate of recurrence (28). Ethanol sclerotherapy has been a suitable replacement for surgery in some patients, especially before IVF. This result poses a question in mind as how HOXA10 and aromatase P450 will change after doing sclerotherapy. It can be a matter of scrutiny in future clinical trials.
